# Telemedicine and Integrated Multidisciplinary Care for Pediatric IBD Patients: A Review

**DOI:** 10.3390/children8050347

**Published:** 2021-04-28

**Authors:** Lauren M. Potthoff

**Affiliations:** 1 Division of Gastroenterology, Hepatology and Nutrition, Pritzker Department of Psychiatry and Behavioral Health, Ann & Robert H. Lurie Children’s Hospital of Chicago, Chicago, IL 60611, USA; lmpotthoff@luriechildrens.org; 2 Department of Psychiatry and Behavioral Sciences, Feinberg School of Medicine, Northwestern University, Chicago, IL 60611, USA

**Keywords:** inflammatory bowel disease, pediatric, telemedicine

## Abstract

The global COVID-19 pandemic brought with it an unprecedented, widespread implementation of telemedicine services, requiring pediatric inflammatory bowel disease (IBD) providers to shift in-person clinic visits to a virtual platform. With the passing of the one-year anniversary of the global pandemic, telemedicine continues to be offered and utilized. Although it remains unclear as to the extent to which telemedicine services will be used in the future, it is critical to understand how integrated multidisciplinary treatment—the standard of care in pediatric IBD—is delivered through a virtual platform. This paper provides an overview of the existing literature examining integrated multidisciplinary care for pediatric IBD provided via telemedicine. The author also presents one integrated multidisciplinary IBD program’s response to the global pandemic and subsequent transition to telemedicine. Challenges around implementation and directions for future research in this area are also discussed.

## 1. Introduction

Inflammatory bowel diseases (IBD) are relapsing remitting conditions characterized by chronic inflammation within the gastrointestinal (GI) tract. IBD is generally classified into two subtypes which are distinguished by the nature of and anatomical location of inflammation: Crohn’s disease (CD), which can involve the entire GI tract, and ulcerative colitis (UC), which is limited to the colon. IBD is associated with a variety of medical and psychosocial complications due to the progressive nature of the disease and often debilitating physical symptoms [1]. The prevalence of pediatric IBDs is on the rise throughout the world [1,2,3,4], with an incidence of 10 per 100,000 children in the United States [5]. While the onset of IBD can occur at any age, it is estimated that 20–30% of IBD cases present prior to age 20 [5].

Compared with healthy peers, children and adolescents with IBD are more vulnerable to increased mental health concerns [6,7], decreased quality of life [8,9,10], and increased psychological distress related to their disease [11]. Youths with IBD are also at an increased risk for poorer academic [8,12], family [13], and social functioning [7]. Furthermore, pediatric IBD is associated with higher disease activity (i.e., more flares), more extensive disease, and a more complicated disease course compared with adult-onset IBD [14,15,16]. Considering the scope of these vulnerabilities, an integrated multidisciplinary care model is increasingly recognized as the gold standard in the treatment of pediatric IBD [17,18,19].

### 1.1. Integrated Multidisciplinary Care and Pediatric IBD

Multidisciplinary treatment is broadly defined as an integrated team approach in which healthcare providers across different disciplines consider relevant treatment options and collaboratively develop comprehensive, individualized treatment plans [20,21]. Within pediatric IBD, this care model often combines the expertise of many providers. While the composition of multidisciplinary pediatric IBD treatment teams vary across institutions, they typically include a combination of IBD gastroenterologists, advanced practice providers, nurses, surgeons, dieticians, psychologists, and social workers in collaborative treatment of the biological, psychological, and social needs of the patient and family throughout the course of the disease [22]. Research specifically examining integrated multidisciplinary treatment approaches within the IBD patient population demonstrates that early intervention and close follow-up from multidisciplinary providers are vital in achieving and maintaining remission [23], minimizing complications [24], identifying existing and acute psychosocial concerns [25] and reducing inpatient and outpatient healthcare costs associated with the disease [26,27,28].

Integrated multidisciplinary care models have typically been implemented during in-person clinic visits. However, with the novel coronavirus disease 2019 (COVID-19) pandemic, the United States Secretary of Health and Human Services released a directive [29] for broad utilization of telemedicine services in an effort to slow the spread of the disease [30]. With this federal directive, providers were required to convert in-person outpatient appointments to a virtual platform.

### 1.2. Telemedicine Overview

Although the terms are often used interchangeably, telemedicine refers to the use of information and communication technologies (ICTs) for remote health care delivery (without direct physical contact with the patient) [31]. In contrast, telehealth employs ICTs in a broader spectrum of clinical activities (e.g., provider-to-provider communications) [32] as well as non-clinical services (e.g., administrative meetings) [33,34].

Prior to the COVID-19 pandemic, telemedicine was fairly limited amongst medical providers. Data from the American Medical Association’s 2016 Physician Practice Benchmark Survey indicate that only 15.4% of physicians in the United States worked in practices that used telemedicine [35], as broad implementation of these services has historically been limited by challenges around reimbursement, licensing, and institutional infrastructure [36,37]. In 2020, widespread adoption of virtual services in response to the global pandemic was possible by several key accommodations, including loosened regulations permitting physicians to offer care across state lines [38], increased reimbursement for visits [39], and relaxed Health Insurance Portability and Accountability Act (HIPAA) standards to accommodate virtual communication platforms [40].

### 1.3. Current Paper

The global pandemic and subsequent public health emergency brought with it an unprecedented, large-scale implementation of telemedicine services. As integrated multidisciplinary care is recognized as a best practice approach for children and adolescents with IBD, it is critical to understand how these services are offered through virtual platforms. The goal of the current paper is to provide an overview of the existing literature examining integrated multidisciplinary care for pediatric IBD patients delivered via telemedicine. For the purposes of this review, telemedicine will only include direct patient-to-provider interactions; discussion of the broader category of digital health platforms in pediatric IBD (i.e., smartphone applications for symptom and disease activity tracking, online support forums) is outside the scope of this article. Additionally, the author will present one IBD program’s response to the COVID-19 pandemic, transition to telemedicine, and describe how multidisciplinary team members continued to provide patient care during this time.

## 2. Telemedicine in Pediatric IBD

The last decade has shown increased efforts at implementing telemedicine for adult patients with IBD [41] as virtual services have the potential to reduce costs associated with managing this disease and increase the availability of specialty care [34]. Within this population, telemedicine is well-accepted and associated with efficient care delivery [41], decreased rates of hospitalizations, improved patient quality of life [42], and increased patient satisfaction, empowerment, and collaboration amongst multidisciplinary providers [43,44]. Use of telemedicine in pediatric IBD care, however, is more limited even among pediatric gastroenterology programs with existing telemedicine services. Prior to the COVID-19 pandemic, an integrated health care delivery system—Kaiser Permanente Northwest Pediatric Gastroenterology—offered a robust telemedicine practice with over half of pediatric gastroenterology visits conducted virtually from 2019 to 2020. However, telemedicine was used less frequently for patients with IBD. Face-to-face visits were more commonly assigned for these patients due to the complicated nature of the disease or for visits requiring multidisciplinary care [45].

Providing clinical care via telephone only (without video) offers many benefits, as this service is found to be convenient, cost-effective, and associated with improved quality of patient care and reduced no-show rates [46,47,48,49]. Especially important for children and adolescents with IBD who often require immediate access to healthcare professionals, telephone clinics and consultations can also facilitate streamlined services [50]. Akobeng et al., (2015) conducted a randomized controlled trial comparing telephone consultations with traditional face-to-face services for pediatric IBD management. Eighty-six patients ages 8–16 were randomized to telephone or in-person services. Patients in the telephone group reported quality of life scores consistent with patients who received in-person care. Further, results indicate that care delivered via telephone reduced consultation times as well as cost of service [51].

## 3. Integrated Multidisciplinary Care for Pediatric IBD and Telemedicine

Existing literature on integrated multidisciplinary visits for pediatric IBD patients delivered via telemedicine is extremely limited both in terms of studied outcomes/benefits as well as descriptive papers on the creation of this line of service. No integrated multidisciplinary IBD programs have published outcome data on the implementation of virtual multidisciplinary clinic appointments either prior to or during the global pandemic. In their letter to the editor of the Journal of Pediatric Gastroenterology and Nutrition, Verstrate et al., (2020) shared how they expanded existing telemedicine capabilities within their integrated multidisciplinary IBD program to continue providing comprehensive care and meeting the diverse needs of this patient population during the global pandemic. It appears that multidisciplinary clinic visits were conducted virtually as detailed in one of their goals for implementing broader telemedicine services (i.e., “Routine care to our patients with IBD, including multidisciplinary visits…,”) yet, no data are published from this program [52].

While it is understood that integrated multidisciplinary programs for pediatric IBD were required to convert appointments from in-person to telemedicine with the onset of the global pandemic, few data are available on the implementation and outcomes of these services. As we await published data on the broad implementation of integrated multidisciplinary telemedicine care for pediatric IBD, one program’s approach to this transition is presented and discussed.

## 4. IBD Program at Ann and Robert H. Lurie Children’s Hospital of Chicago

### 4.1. Program Overview

Officially established in 2015, the integrated multidisciplinary IBD program at Ann and Robert H. Lurie Children’s Hospital of Chicago treats over 600 patients and is staffed by four IBD gastroenterologists, two advanced practice providers (APP), three nurses (RN), a nurse coordinator, two dieticians, a social worker, a psychologist, a medical assistant, a clinical research coordinator, and an administrative program coordinator. Using this multidisciplinary team approach, the program provides comprehensive diagnostic evaluation and treatment for patients from infancy through adolescence experiencing all types of IBD, including CD, UC, IBD-unclassified, very early onset IBD, and IBD secondary to immune-defective/deficient disorders.

Patients are referred to the IBD program from internal providers within the GI division (inpatient and outpatient referrals) as well as providers outside the hospital system. Regardless of referral source, patients are seen by a provider in the program within one to four weeks of diagnosis. Patients are primarily followed by an IBD gastroenterologist, APRN, or PA. Frequency of appointments with primary IBD providers is determined by the patient’s current disease. Patients with new IBD diagnoses, those experiencing a worsening of symptoms, or implementing a new treatment regimen can be seen as often as several times a month. Typically, well patients are seen once every three to six months, depending on current treatments and pubertal status. As Lurie Children’s IBD program is large in terms of providers as well as patients, frequent provider-to-provider communication is necessary in the coordination and provision of comprehensive care.

### 4.2. Coordination of Multidisciplinary Care

At the beginning of each week, the multidisciplinary team participates in a clinic planning meeting. During this meeting, the team discusses and reviews the needs of patients who have clinic appointments that week. As the IBD program treats a diverse group of patients in terms of age, severity of disease, and socioeconomic status, the needs of patients and families vary greatly and often require the expertise of different providers at different points during care. During the weekly meetings, patients new to the program are identified and multidisciplinary providers plan to meet with the patient and family during their clinic appointment to introduce themselves and the services they offer. A provider may also identify an existing patient during the meeting who requires additional supports around, for example, adjusting to a diagnosis, medication or dietary adherence, mood or anxiety concerns, or social determinants of health that may impact care (i.e., reliable transportation, insurance issues). With this identification, the respective team member will meet with the patient and family at his or her upcoming appointment. Alternatively, as team members are physically present during clinic, support is also offered through an outpatient consultation model. For example, the primary provider may request that the psychologist consult with a patient who reported newly onset anxiety symptoms during the clinic visit. As concerns can occur acutely in this patient population, some patient needs may not have been previously known during the clinic planning meeting. In each of these scenarios, respective members of the multidisciplinary team meet with the patient and family to discuss relevant concerns and coordinate follow-up with that provider, as needed.

As Lurie Children’s IBD program continues to grow, new initiatives and advances in patient care are regularly discussed. A dedicated IBD social worker and psychologist were recently added to the program. These providers are motivated to implement psychosocial standards of care that are consistent with integrated multidisciplinary pediatric IBD programs at other institutions. For example, these providers are in the early stages of integrating routine psychosocial screenings to clinic visits as well as piloting a program assisting adolescent patients in preparation for transition to adult IBD care.

### 4.3. Program Response to COVID-19 Pandemic

As Lurie Children’s IBD program exclusively offered in-person clinic appointments prior to the global pandemic, the program’s response to virtual care occurred in stages. To facilitate provider-to-provider communication, telehealth was immediately implemented in accordance with the state of Illinois’ and city of Chicago’s mandated stay-at-home orders issued on 21 March 2020 [53]. The team continued to hold weekly clinic planning meetings through a secure conferencing platform. While the team awaited institutional credentialing for the HIPAA-compliant telemedicine software to conduct video outpatient appointments with patients, providers began conducting appointments via telephone. Once the providers obtained approval for telemedicine encounters, the team transitioned to video appointments.

The program utilized two different models when providing multidisciplinary care via telemedicine: synchronous and asynchronous appointments. The majority of clinic appointments were asynchronous in that separate telemedicine appointments were scheduled with different providers. While separate encounters, appointments were scheduled as closely together as possible. For example, if a patient required nutritional support, he or she would meet with the dietician within a couple of days of their clinic appointment with the IBD provider. Occasionally, providers were able to coordinate schedules and conduct telehealth appointments synchronously. In these instances, the patient and family would initially meet with the primary IBD provider and then, following completion of that portion of the appointment, the next provider would sign onto the same telemedicine appointment. As Lurie Children’s IBD program consists of many providers, it is important to understand how different team members functioned during the global pandemic in their use of telemedicine.

### 4.4. IBD Gastroenterologists and Advance Practice Providers

As mentioned above, these providers initially began conducting clinic appointments by telephone. They utilized their existing clinic templates and saw patients via video for regular clinic appointments as the platform for video appointments became available.

### 4.5. RNs and Nurse Coordinator

The RNs operated asynchronously with primary IBD providers by completing nursing intakes prior to the patient’s clinic appointment. As they were not present during the virtual appointment, RNs completed their intake note in the electronic medical record for the IBD provider to review before meeting with the patient and family. Additionally, RNs continued to monitor patients’ electronic medical charts (i.e., patient/parent messages, lab results) and communicated with IBD providers via phone or secure message in continued coordination of care. In the event of an acute concern or emergency, RNs alerted IBD providers immediately. Any required forms for the clinic visit were sent electronically to families either via secure email or medical chart.

### 4.6. Dieticians

With the implementation of video telemedicine appointments, the dieticians were able to complete nutritional assessments virtually. While nutrition-focused physical exams were limited by the telemedicine model, providers were able to efficiently provide nutrition counseling and education during these appointments. Dieticians were able to work with families in providing specific recommendations and guidelines as well as identifying and working towards short-term goals. Anecdotally, the dieticians shared that providing care via telemedicine was beneficial in that it allowed them to see into patients’ homes and identify factors that could contribute to nutrition management. Dieticians also noted that with this shift in perspective, families endorsed feeling empowered to make changes in diet, as providers used the pandemic as an opportunity to encourage families to engage in additional opportunities for healthy eating and wellness. These appointments were also conducted asynchronously with the patient’s primary IBD provider. The dietician does not bill for services rendered during multidisciplinary IBD clinic.

### 4.7. Psychologist

Consistent with other providers, the psychologist initially conducted telemedicine visits with patients via telephone who requested to keep scheduled appointments despite limitations around meeting in person. Appointments were transitioned to video once credentialing was approved. The psychologist completed visits both synchronously and asynchronously with IBD providers. Synchronous visits were conducted the same day as the patients’ visit with the gastroenterologist or APPs. Asynchronous visits were scheduled within the same week as the medical appointment. As routine psychosocial screening procedures have not yet been launched in this clinic, recommendations for which patients should meet with psychology continued to be discussed during weekly clinic planning meetings. Regarding billing, although the dietician and social worker do not complete billing during multidisciplinary clinics, the psychologist does. As the psychologist is offering interventions and supports specifically related to the patient’s medical condition, health and behavior billing codes are used rather than psychotherapy CPT codes.

### 4.8. Social Worker

Compared with other providers on the team, the IBD social worker faced unique challenges in utilizing telemedicine. The IBD social worker does not have a template for appointments, therefore she does not schedule nor bill for her interactions with patients and families. During the peak of the global pandemic and mandated stay-at-home order, the social worker conducted follow-ups with families via telephone. These follow-up calls were often extensive, offering various levels of support and resources. Although there was no option for the IBD social worker to meet with patients and families via video, this telemedicine model facilitated flexibility in maintaining connections with families.

### 4.9. Clinical Research Coordinator

While unable to meet with study participants in person, the clinical research coordinator consented participants via secure phone portal approved through the institution’s IRB to ensure confidentiality. Using virtual communication platforms, she was also able to complete study initiation, closeout, monitoring, and site selection visits. To facilitate these virtual interactions, she securely sent study documents prior to the scheduled meeting. Additionally, the clinical research coordinator continued to complete prospective and retrospective chart reviews. Although meetings with principal investigators or study team members were not held in person, she was able to interact with these individuals virtually, as well, in order to resolve questions remotely.

### 4.10. Medical Assistant and Administrative Program Coordinator

As these team members typically interact with patients and families via phone or electronic medical record messaging, they were able to continue in their roles generally unaltered in the context of the global pandemic. In addition to scheduling patients for clinic, the administrative program coordinator also sent out appointment links for clinic visits through the HIPPA-compliant telemedicine platform.

### 4.11. Telemedicine Pitfalls

This IBD program’s transition to telemedicine visits in response to the COVID-19 global pandemic was not without its pitfalls and challenges. First, transitioning a fully, in-person multidisciplinary clinic to telemedicine added to the workload of several team members. The administrative program coordinator was tasked with offering technological support to families in downloading and using the HIPAA-compliant software in addition to sending out appointment links for each visit. As is expected with any advent of technology, technological issues were unfortunately reported. Occasionally, families would experience Internet/Wi-Fi connectivity issues, limiting the quality of the video and audio during the appointment, or challenges around logging into the platform and locating the appropriate link for the visit. During these times, team members collaborated as much as possible to assist the family but, unfortunately, there were occasions during which families were unable to attend clinic visits due to these technological issues. In these instances, clinic appointments were rescheduled for the next soonest availability with providers. Unsurprisingly, issues around technology created dissatisfaction for providers as well as patients and families.

With asynchronous multidisciplinary telemedicine visits, additional communication amongst team members was required, subsequently adding to their respective workloads. During in-person clinics, team members are typically located in the same team space within clinic, facilitating effective communication amongst providers throughout the clinic day. With asynchronous telemedicine visits, providers were required to communicate via phone, secure email, or via the electronic medical record to share pertinent information regarding the patient and that provider’s visit. While this decreased the ease with which team members communicated, collaboration was not lost as providers remained committed to the multidisciplinary approach to care and ensuring that all team members were informed of relevant concerns.

Despite these challenges and limitations, the general, anecdotal consensus from patients and families was appreciation and relief around the ability to continue receiving multidisciplinary IBD treatment while exercising COVID-19 precautions. As this clinic had not conducted telemedicine visits prior to the global pandemic, there was of course a learning curve inherent with the launching and utilization of a fully virtual multidisciplinary clinic. However, team members reported that they were still able to provide the quality care that patients and families had come to expect. This is likely attributable to several factors, including this specific clinic population’s tendency to communicate regularly with team members via the medical chart around symptoms, concerns, and lab results as well as the strong multidisciplinary team culture and workflow that existed prior to the global pandemic.

### 4.12. Summary

This is an example of how one integrated multidisciplinary pediatric IBD program transitioned from in-person to telemedicine visits at the onset of the global pandemic. Although this transition was met with some challenges (i.e., waiting for credentialing) and discussed pitfalls, the benefits associated with telemedicine visits are obvious. First, the team was able to continuously provide multidisciplinary care with minimal disruption from the global pandemic. While awaiting credentialing, providers continued to communicate with families and conduct appointments via telephone. While the majority of multidisciplinary visits occurred asynchronously, families and providers communicated the benefit of this model. In-person clinic visits within this program can last up to three hours depending on the number of providers needing to meet with the patient and family. With asynchronous visits, appointments were briefer. Some providers shared a preference for the asynchronous model as they observed families to be more receptive to information provided during a separate visit rather than at the end of an already hours-long appointment.

As providers continue to navigate through the global pandemic, the future of telemedicine use in this program remains unclear and dependent upon many factors (i.e., insurance reimbursement). However, team members continue to offer telemedicine services for patients and families who communicate this preference. Multidisciplinary clinic visits also continue to be offered and conducted virtually although at a reduced frequency as in-person clinic visits have been resumed. Additionally, some providers have expressed interest in implementing and offering additional services to patients and families through a virtual platform. The IBD social worker shared a vision for conducting separate, virtual sessions with families around transitioning to adult care. Telemedicine also provides increased opportunities for the IBD social worker and psychologist to conduct psychosocial support groups for both patients and families. As attendance in therapy and support groups has historically been inconsistent [54], offering this service virtually may result in improved participation.

## 5. Discussion

Telemedicine is recognized as a quality method of healthcare delivery that is also efficient and cost effective [33,55]. In response to the COVID-19 global pandemic, healthcare systems implemented a rapid expansion of telemedicine services as most clinical care became exclusively virtual. These virtual services were effective in providing continued care during a time in which providers were unable to meet with patients face-to-face. While the rollout of the COVID-19 vaccine has permitted providers to resume in-person clinic visits, many believe that telemedicine services will continue to be offered and utilized [56,57,58,59]. With the anticipation of continued telemedicine services, it is critical to understand how virtual services can accommodate integrated multidisciplinary care for children and adolescents with IBD.

As discussed, existing research examining the virtual delivery of integrated multidisciplinary care for children and adolescents with IBD is extremely limited. Pediatric gastroenterology programs with existing telemedicine clinics offered virtual care less frequently to IBD patients prior to the global pandemic [45]. Research examining telephone consultations between IBD providers and patients and families for IBD care offered promising results in that patient quality of life is consistent across both groups [51]. The missing piece, however, is the integrated multidisciplinary treatment component.

It is understood that pediatric IBD programs offering integrated, multidisciplinary care were required to transition services to a virtual platform in response to the global pandemic [52]. Yet, at the time of this writing, no literature exists on the virtual rollout of these programs, teams’ approach to providing multidisciplinary care via telemedicine platforms, or patient or disease outcomes. In response to this dearth of literature, the author provided an in-depth example of the approach utilized by one integrated multidisciplinary pediatric IBD program, highlighting the roles of multidisciplinary team members and how they continued to provide care to patients during this unprecedented period.

## 6. Limitations

In the absence of literature on integrated multidisciplinary pediatric IBD treatment delivered via telemedicine, the author presented one team’s approach to adapting in-person multidisciplinary clinic visits to a telemedicine model. The example provided in this article reflects one integrated multidisciplinary IBD program’s response to the adoption of telemedicine in response to the COVID-19 pandemic and thus precludes full generalizations to programs at other institutions. However, it is the author’s hope and intent that readers can identify relevant aspects in the description of virtual multidisciplinary care provided for this population.

## 7. Directions for Future Research

There are many opportunities and directions for future research. First, as telemedicine services continue to be offered, it is important to understand the extent to which virtual services are currently utilized among children and adolescents with IBD, specifically. Further, as integrated multidisciplinary treatment is accepted as the standard of care for pediatric IBD, specific attention should be afforded to the virtual application of this treatment model. Future research in this area should examine the creation and implementation of virtual integrated multidisciplinary IBD clinics with a focus on structure and virtual clinic workflow (i.e., synchronous vs. asynchronous appointments), as well as patient and medical outcomes (i.e., patient and family satisfaction, quality of life, psychosocial functioning, rate of hospitalization). It is critical to understand whether medical outcomes among children and adolescents who receive their IBD care virtually are consistent with their counterparts receiving in-person multidisciplinary care. Finally, many digital health platforms exist to support pediatric IBD patients. While outside the scope of this paper, smartphone applications that support disease and symptom tracking have been found to be successful, particularly for adolescent patients [60,61]. Future research should examine how multidisciplinary care can be utilized through these digital platforms as well.
